# Stressing the Importance of Uplifts: The Associations Among Stressors, Uplifts, Health Problems and Subjective Age

**DOI:** 10.1093/geroni/igaf122.1490

**Published:** 2025-12-31

**Authors:** Dwight Tse

**Affiliations:** University of Strathclyde, Glasgow, Scotland, United Kingdom

## Abstract

Subjective age refers to the age a person feels and is linked to healthy aging. Studies suggest stressors and uplifts influence subjective age, although the mechanisms remain relatively unclear. Given that health problems are linked to feeling older and vary daily, this study examines the indirect effects of daily stressors and uplifts on subjective age through daily health problems (DHPs). Using a 14-day diary design, 85 UK participants aged 50-85 completed a baseline survey and daily questionnaires on stressors, uplifts, health symptoms, and felt age, yielding 933 entries (78% completion). Multilevel mediation analysis results showed that within individuals, more stressors and fewer uplifts predicted more DHPs and older subjective age, with significant indirect effects of stressors and uplifts on subjective age through DHPs. At the between-person level, uplifts—but not stressors—predicted lower DHPs and, in turn, younger subjective age. Compared to daily stressors, uplifts consistently supported healthier aging at both levels, highlighting the short-term and long-term benefits of everyday positive events. In parallel with the subjective weathering hypothesis, uplifts may initiate a “fortification” process that helps older adults maintain and enhance well-being and functioning.

